# Patellofemoral pain syndrome based on biomechanical monitoring and intervention: a single-center, prospective, interventional cohort study

**DOI:** 10.3389/fsurg.2026.1843017

**Published:** 2026-06-01

**Authors:** Huiwu Zhang, Bei Liu, Daren Zhao, Lei Liu, Junrong Chen, Jian Yang, Jingping Wang, Yulong Qin, Zhongzheng Hu

**Affiliations:** 1 Center for Sports Medicine, Sichuan Provincial Orthopedics Hospital, Chengdu, China; 2 Department of Society and Health, Faculty of Social Sciences and Humanities, Mahidol University, Salaya, Nakhon Pathom, Thailand

**Keywords:** biomechanical monitoring, cohort study, exercise therapy, intervention, patellofemoral pain syndrome

## Abstract

**Introduction:**

Patellofemoral pain syndrome (PFPS) is a prevalent chronic knee disorder that poses significant clinical challenges due to its high incidence and suboptimal long-term outcomes. Current diagnostic approaches, primarily reliant on static imaging and subjective assessment, fail to capture the underlying dynamic biomechanical dysfunction. To address this gap, this study integrates dynamic radiography with three-dimensional gait analysis to construct a dynamic biomechanical model of the knee joint. We will quantify key parameters such as patellar tracking. The research is designed to achieve three core Objectives: firstly, to quantify dynamic biomechanical parameters in PFPS patients using dynamic DR and gait analysis; secondly, to compare the effects of biomechanically monitored targeted exercise combined with TCM vs. conventional exercise combined with TCM on pain and function; thirdly, to develop a biomechanics-guided clinical rehabilitation protocol for PFPS patients.

**Methods and analysis:**

This single-centre, prospective, interventional cohort study will investigate the effectiveness of a biomechanically monitored targeted exercise protocol combined with Traditional Chinese Medicine (TCM) compared with conventional exercise combined with TCM in patients with patellofemoral pain syndrome (PFPS). We will consecutively recruit 96 eligible patients. Participants will be assigned to either an experimental group or a control group based on their preference after receiving standardized information. The experimental group will receive biomechanical monitoring, targeted exercise, and Traditional Chinese Medicine (TCM), whereas the control group will receive conventional exercise and the same TCM regimen.

Outcomes will be assessed at baseline, 6 weeks, 6 months and 3 years post-intervention. The primary outcome measure will be pain intensity measured using the Visual Analog Scale (VAS). Secondary outcome measures will include: biomechanical parameters; pain (Knee Pain Scale); joint function (Lysholm Knee Scoring Scale); kinetic parameters (mean power and pulling acceleration during stair climbing using the IDEEA system); spatiotemporal gait parameters (walking speed, step frequency, double support time during stair climbing using IDEEA); and quality of life (36-Item Short Form Health Survey).

For statistical analysis, independent samples *t*-tests will be used for between-group comparisons, and linear mixed-effects models will be applied for outcomes measured at multiple time points. To control for expectation bias, patients' treatment expectation scores will be recorded at baseline and adjusted as covariates if significant between-group differences exist. Baseline characteristics will be compared, and any imbalanced variables will be included as covariates in the primary analysis. A two-tailed *P*-value < 0.05 will be considered statistically significant.

**Clinical Trial Registration:**

Chinese Clinical Trial Registration Number: ITMCTR2025002287

## 1 Introduction

Patellofemoral pain syndrome (PFPS) is the most prevalent chronic knee disorder among adolescents and adults, characterized primarily by peripatellar or retropatellar pain during patellofemoral joint loading activities (e.g., squatting, stair ambulation, and running) (1–6). Evidence indicates that about 80% of individuals with PFP continue to have pain despite completing rehabilitation programs (7). Patellofemoral pain syndrome represents a major socioeconomic burden, evidenced by musculoskeletal disorder costs of £7.4 billion per year in the UK and over $213 billion in the US (5). Therefore, this disease presents a critical clinical challenge, driven by the convergence of high prevalence, suboptimal long-term outcomes and significant economic costs that highlight the pressing need for objective diagnostic tools.

It has been established in the literature that the diagnosis of patellofemoral pain is challenging, partly due to the absence of a clear gold-standard reference test (8, 9). Within the domain of medical imaging, a key limitation is that static imaging cannot adequately assess dynamic patellar function, as it provides only a snapshot of non-physiologic joint position (10). Consequently, clinical decision-making becomes disconnected from the core pathomechanism, resulting in imprecise diagnosis, non-targeted therapy, and unstable therapeutic outcomes.

To address these challenges, it is imperative to shift the focus toward dynamic biomechanical assessment. This approach directly targets the core issue by quantifying patellofemoral joint function during activity, thereby bridging the critical gap left by conventional subjective evaluation and static imaging.

Research indicates that the etiology of PFPS is multifactorial, with biomechanical factors such as quadriceps weakness constituting a key modifiable risk factor (4). Biomechanical monitoring technologies offer a means to directly quantify the dynamic function of the patellofemoral joint. These techniques enable the objective measurement of kinematic and kinetic parameters during functional activities such as walking, running, stair ascent/descent, and squatting, thereby uncovering the core pathological mechanism of PFPS: dynamic biomechanical dysfunction. Biomechanical monitoring technologies provide critical technical means for the accurate assessment of PFPS. The core techniques primarily include three-dimensional motion capture systems (11, 12), force platforms (13, 14), and surface electromyography (15–17), all of which have been widely applied in relevant research.

Additionally, a review of the literature reveals that the core advantage of dynamic DR over traditional static DR lies in its ability to capture functional and dynamic anatomical information of the knee joint under weight-bearing or motion conditions (18). This enables earlier and more comprehensive detection of pathological changes that manifest only under load or during activity. Consequently, it significantly enhances the diagnostic capability for functional knee disorders, such as patellar tracking abnormalities and early joint space narrowing. Future trends will focus on integrating dynamic DR, gait analysis technology, and imaging data (19). By combining joint motion trajectories, mechanical loading, and three-dimensional imaging parameters, an integrated “imaging manifestation-biomechanical mechanism” database will be established. This will further elucidate the pathophysiological processes of the disease and optimize surgical planning, such as the personalized design of tibial tubercle osteotomy and medial patellofemoral ligament reconstruction.

This study focuses on patellofemoral pain syndrome (PFPS) and aims to integrate dynamic DR and gait analysis technology to construct a dynamic three-dimensional biomechanical model of the knee joint. Current diagnostic approaches, primarily reliant on static imaging and subjective assessment, fail to capture the underlying dynamic biomechanical dysfunction. However, PFPS is intrinsically related to abnormal joint loading during dynamic activities such as stair climbing and squatting. Therefore, to provide a rational basis for individualized rehabilitation, biomechanical monitoring is the key to targeted exercise therapy, as it offers precise information on individual movement patterns and joint loading. This study aims to achieve three core objectives: firstly, to quantify dynamic biomechanical parameters in patients with patellofemoral pain syndrome (PFPS) using dynamic DR and gait analysis; secondly, to compare the effects of biomechanically monitored targeted exercise combined with Traditional Chinese Medicine (TCM) vs. conventional exercise combined with TCM on pain and function in patients with PFPS; thirdly, to develop a biomechanics-guided clinical rehabilitation protocol for patients with PFPS.

## 2 Methods and analysis

### 2.1 Study design

This study will be prospective, interventional cohort study conducted at a single center.

### 2.2 Setting

This study will be conducted at Sichuan Provincial Orthopedics Hospital in China. The hospital holds the distinction of being China's first sports medicine hospital and a leading Grade A Tertiary specialized orthopedic center that integrates traditional Chinese and Western medicine. The study is scheduled to begin in June 2026, with completion of the initial phase targeted for December 31, 2026. A planned three-year follow-up period for all participants will commence thereafter. Follow-up will be divided into short-term and mid- to long-term assessments. Re-examination and follow-up will be conducted at 6 weeks, 6 months, and 3 years post-intervention.

### 2.3 Study population

#### 2.3.1 Recruitment

Prospective participants will be identified and recruited from the outpatient clinics of the Sichuan Provincial Orthopedics Hospital. The target enrollment is 96 patients with a diagnosis of patellofemoral pain syndrome (PFPS).

### 2.4 Eligibility criteria

#### 2.4.1 Inclusion criteria

To be eligible, individuals must (1) be 40 to 60 years of age; The age range of 40–60 years was selected based on a 10-year review of hospital medical records showing that PFPS patients in our setting are predominantly concentrated in this age group. (2) fulfill the clinical diagnostic criteria for patellofemoral pain syndrome (PFPS), including anterior knee pain exacerbated by functional activities (e.g., stair negotiation, squatting), with imaging (x-ray or MRI) confirming no other knee pathologies; and (3) provide written informed consent.

#### 2.4.2 Exclusion criteria

Individuals will be excluded if they have (1) significant systemic disease (e.g., cardiovascular, pulmonary, hepatic, renal, or psychiatric) that could confound assessment or outcomes; (2) concomitant major knee joint pathology such as fractures, ligamentous ruptures, rheumatoid arthritis, or gouty arthritis; or (3) undergone any recent surgical procedure or targeted therapy for the patellofemoral joint. (4) radiographic evidence of knee osteoarthritis with Kellgren-Lawrence grade≥2.

### 2.5 Group allocation and intervention

Patients will be assigned to either the experimental group or the control group based on their preference after receiving standardized information about both interventions. Specifically, the experimental group will receive biomechanical monitoring, targeted exercise, and Traditional Chinese Medicine (TCM), whereas the control group will receive conventional exercise and the same TCM regimen as the experimental group.

### 2.6 Sample size

The sample size for this study was determined through an *a priori* power analysis based on a two-independent-sample means comparison, aimed at detecting differences in the change scores (pre- to post-intervention) of the primary continuous outcome (VAS) between the two groups. Based on previous studies or pilot data, we assumed a mean difference of *δ* = 1.1*σ* between the groups, with an estimated population standard deviation of *σ* = 2.0. The significance level was set at *α* = 0.05 (two-tailed) and the statistical power (1–*β*) at 0.95. Using the corresponding formula (1), a sample size of 43 participants per group was calculated, resulting in a total of 86 participants.

$$n_{1}=n_{2}=2{\left(\frac{z_{\alpha /2}+z_{\beta }}{\delta /\sigma }\right)}^{2}$$

To account for potential loss to follow-up or dropouts and to ensure reliable between-group comparisons and data analysis in a real-world setting, the sample size was increased by approximately 10%. Thus, a total of *n* = 96 participants with patellofemoral pain syndrome are planned to be recruited, with *n* = 48 in each group. All eligible patients will be consecutively assessed for eligibility during the study period and assigned to the corresponding intervention group.

### 2.7 Study groups and interventions

Eligible participants will be allocated to one of two groups.

### 2.8 Experimental group

This group will receive biomechanical monitoring, targeted exercise therapy, and Traditional Chinese Medicine (TCM) treatment.

### 2.9 Biomechanical monitoring

The biomechanical monitoring in this study consists of two complementary components: a laboratory-based multi-technology assessment integrating CBCT, dynamic DR, MRI, and gait analysis, and a real-world wearable sensor assessment using the IDEEA system during stair climbing.

### 2.10 Part 1: Laboratory-based multi-technology assessment

The system integrates four core technologies: Cone-Beam CT (CBCT) provides high-precision static three-dimensional bone structure; dynamic DR enables continuous imaging capture of knee joint flexion/extension movement; MRI clearly displays the morphology of soft tissues such as cartilage and ligaments; and the gait analysis system simultaneously records motion trajectories and surface electromyography (sEMG) signals, supplementing data from the perspectives of dynamics and muscle activity.

#### 2.10.1 Workflow

Subjects undergo the aforementioned examinations while performing standardized motions (e.g., stepping up/down). Dynamic DR, gait analysis, and sEMG recording will be performed synchronously during the same standardized motion. Dynamic DR and gait analysis record the continuous movement process. A brief pause is made at the angle where symptoms are most pronounced to perform CBCT scanning, thereby acquiring precise three-dimensional data of this key posture.

#### 2.10.2 Data fusion and modeling

The core of data processing lies in multi-source information fusion. A preliminary skeletal motion model is established based on the dynamic DR image sequence. This model is then registered and corrected using the high-precision static CBCT images to enhance its geometric accuracy. Finally, sEMG data is introduced to validate the biomechanical plausibility of the model's motion. Through iterative optimization, a three-dimensional motion model is ultimately constructed that can authentically reflect the biomechanical characteristics of the patellofemoral joint under dynamic loading. All biomechanical measurements will be performed by the same senior technician, and equipment will be regularly calibrated to ensure reliability.

### 2.11 Part 2:Wearable device assessment (IDEEA)

In addition to laboratory-based measurements, a wearable micro-inertial sensor system (IDEEA, Intelligent Device for Energy Expenditure and Activity) will be used to quantify lower limb kinematics in a real-world setting. The IDEEA system used in this study is manufactured in China and provided by Hefei Minglisun Electronics Co., Ltd. (brand: MiniSun). The IDEEA assessment is conducted separately in a real-world stair climbing environment and is not synchronized with the laboratory-based measurements (20). The IDEEA system uses high-frequency multi-sensor fusion (accelerometer and gyroscope) to record spatiotemporal parameters including walking speed, step frequency, and double support time, as well as kinetic characteristics such as mean power and pulling acceleration (21).

#### 2.11.1 Testing procedures

Participants will wear the IDEEA system while performing a continuous stair climbing test on a standard architectural staircase (step height: 14 cm, depth: 27 cm, slope: approximately 27.4°, 18 steps with a mid-stair landing) (22). Under no external verbal interference and without using handrails, participants will ascend the 18 steps at their natural walking pace without stopping, for three consecutive trials (22).

#### 2.11.2 Data extraction

Gait parameters during stair climbing will be exported using the IDEEA system software, and the arithmetic mean of the three trials will be used for statistical analysis. The following parameters will be extracted: kinetic parameters including mean power (W) and pulling acceleration (g); and spatiotemporal gait parameters including walking speed (m/s), step frequency (steps/min), and double support time (ms).

#### 2.11.3 Rationale for stair climbing as a test activity

Stair climbing is a high-load daily activity that requires the knee joint (particularly the patellofemoral joint) to generate and withstand significant extensor moments, leading to high quadriceps activation and increased patellofemoral joint pressure (23). This high mechanical load makes stair climbing a key activity that induces and exacerbates pain in patients with patellofemoral pain syndrome. Among various daily tasks, stair climbing induces the most severe knee pain, followed by stair descending and level walking, highlighting its unique clinical value in assessing symptomatic burden (24). Studies have also shown that abnormal increases in peak knee flexion moment and valgus moment during stair climbing are closely associated with cartilage degeneration and are important mechanical indicators for predicting disease progression (25).

### 2.12 Targeted exercise therapy

A personalized exercise protocol is developed based on the patient's clinical status and physical capacity. The protocol comprises periarticular knee muscle strengthening and joint flexibility exercises to enhance lower extremity stability and optimize knee joint load distribution. Strengthening exercises target the vastus medialis obliquus (VMO) and gluteal muscles, specifically including VMO setting and supine bridging. Flexibility exercises are designed to stretch the tensor fasciae latae (TFL), gluteus medius, hamstrings, and gastrocnemius, specifically including supine cross-body stretch and seated hamstring stretch. Each session lasts 30–60 min and is performed twice daily (morning and evening) over a 6-week period, as 6 weeks is the minimum effective duration and compliance dropped after 6 weeks in our pilot data. The specific interventions are described below.

#### 2.12.1 Vastus medialis obliquus (VMO) setting (right side as example)

Position: Supine. The left knee is flexed to 90° with the left foot placed flat on the bed. The right leg is extended. The participant contracts the quadriceps, dorsiflexes the ankle, and externally rotates the foot to 30°. The right lower extremity is then elevated until the heel clears the bed surface by 30 cm. The position is held for 10 s, followed by a controlled lowering phase over 10 s (one repetition). A 5 s rest is allowed between repetitions. Ten repetitions constitute one set. Three sets are performed per session, with a 1 min rest interval between sets. Frequency: Once daily.

#### 2.12.2 Supine bridge (gluteal strengthening)

Position: Supine with both knees flexed and feet placed flat on the bed, shoulder-width apart. The arms rest along the trunk. The participant inhales, contracts the abdominal and gluteal muscles, and lifts the pelvis by extending the hips until the shoulders, hips, and knees are aligned in a straight line. Core stability is maintained throughout the movement. The end position is held for 10 s, followed by a controlled descent back to the starting position. Ten repetitions constitute one set. Three sets are performed per session, with a 1 min rest interval between sets. Frequency: Once daily.

#### 2.12.3 Supine cross-body stretch (right side as example)

Position: Supine. The right hip and knee are each flexed to 90°. The right hand grasps the edge of the bed to stabilize the right shoulder and prevent ipsilateral shoulder elevation. The lumbar spine remains in contact with the bed surface as much as possible. The left hand is placed on the distal right thigh, and a cross-body stretching maneuver is performed until a sensation of tension is perceived in the right gluteal region and lateral right thigh. The stretch is held for 20 s. The procedure is repeated on the contralateral side. Five repetitions are performed per side. Frequency: Once daily.

#### 2.12.4 Seated hamstring stretch (right side as example)

Position: Seated with the trunk erect. The right leg is fully extended. The participant shifts the body weight forward and flexes the trunk forward until a stretching sensation is felt along the posterior aspect of the right thigh. The stretch is held for 20 s. The procedure is repeated on the contralateral side. Five repetitions are performed per side. Frequency: Once daily.

### 2.13 TCM treatment

#### 2.13.1 Oral TCM treatment tailored by syndrome differentiation

For wind-cold-dampness obstruction with blood stasis: Administer Xiao Zeng Qiang Gu Pian (hospital preparation, approval number: Chuan Yao Zhi Bei Zi Z20190330000, 0.25 g/tablet. Main ingredients: Rehmanniae Radix Praeparata, Pyrolae Herba, Cistanches Herba, Spatholobi Caulis, Drynariae Rhizoma, Cibotii Rhizoma, Angelicae Pubescentis Radix, Erythriniae Cortex, Crataegi Fructus (scorch-fried), Massa Medicata Fermentata (scorch-fried), Hordei Fructus Germinatus (scorch-fried). Main efficacy: Tonifies the liver and kidney, dispels wind-dampness.). Dosage: 4 tablets each time, three times daily. Also administer Leng Xi Kou Fu Ye (hospital preparation, approval number: Z20190335000, 10 ml/vial. Main ingredients: Atractylodis Macrocephalae Rhizoma, Morindae Officinalis Radix, Poria, Saposhnikoviae Radix, Cyperi Rhizoma, Achyranthis Bidentatae Radix, Dendrobii Caulis, Dioscoreae Hypoglaucae Rhizoma. Main efficacy: Dispels wind-dampness, strengthens tendons and bones.). Dosage: 10 ml each time, three times daily.

For cases predominantly characterized by wind-damp-heat obstruction with blood stasis: Administer Chuang Shang Xiao Zhong Pian (hospital preparation, approval number: Z20190333001, 7.2 g/box. Main ingredients: Notoginseng Radix et Rhizoma, Panacis Japonici Rhizoma. Main efficacy: Dispels stasis, stops bleeding, reduces swelling, alleviates pain.). Dosage: 3 tablets each time, three times daily. Also administer Xiao Zeng Qiang Gu Pian (as above). Dosage: 4 tablets each time, three times daily, to tonify the liver and kidney, activate blood circulation, and resolve stasis. Oral treatment lasts 6 weeks. Non-steroidal anti-inflammatory drugs (NSAIDs) may be administered orally if necessary.

#### 2.13.2 External TCM treatment tailored by syndrome differentiation

For wind-cold-dampness obstruction with blood stasis syndrome: Apply Xin Shang Xiao Zhong San (hospital preparation, approval number: Chuan Yao Zhi Bei Zi Z20190301000, 50 g/bag. Main ingredients: Phellodendri Chinensis Cortex, Rhei Radix et Rhizoma, Corydalis Rhizoma, Paeoniae Radix Rubra, Angelicae Dahuricae Radix, Sargentodoxae Caulis, Notopterygii Rhizoma et Radix, Angelicae Pubescentis Radix, Vladimiriae Radix, Hibisci Mutabilis Folium, Draconis Sanguis, Menthol, Camphora, Borneolum Syntheticum. Main efficacy: Activates blood circulation to resolve stasis, reduces swelling and alleviates pain.) mixed with Angelicae Dahuricae Radix powder (10 g), Asari Radix et Rhizoma powder (10 g), Carthami Flos powder (10 g), and Arisaematis Rhizoma powder (10 g) for external application.

For wind-damp-heat obstruction with blood stasis syndrome: Apply Xin Shang Xiao Zhong San (as above) mixed with Angelicae Dahuricae Radix powder (10 g), Carthami Flos powder (10 g), Paeoniae Radix Rubra powder (10 g), and Arisaematis Rhizoma powder (10 g) for external application.

Specific method for both: Take equal parts (one-quarter each) of Xin Shang Xiao Zhong San and the mixed herbal powder, add an appropriate amount of honey water, and mix into a paste. Apply the paste onto gauze or foam dressing and affix it to the affected knee. Apply externally for 8–12 h each time, once daily. If allergic reactions such as skin itching or redness occur during application, discontinue use immediately. Continuous external treatment lasts 6 weeks.

## 3 Control group

This group will receive conventional exercise and the same TCM regimen as the experimental group. The conventional exercise consists of quadriceps exercise (right side as example): supine position with the left knee flexed to 90° and the left foot placed flat on the bed, while the right leg is extended. The participant contracts the quadriceps and dorsiflexes the ankle, then elevates the right leg until the heel clears the bed surface by 30 cm. The position is held for 10 s, followed by a controlled lowering phase over 10 s (one repetition), with a 5 s rest between repetitions. Ten repetitions constitute one set, and three sets are performed per session with a 1 min rest interval between sets. Frequency: once daily for 6 weeks.

### 3.1 Outcome measures

#### 3.1.1 Primary outcome

The primary outcome is pain intensity measured using a 0–10 Visual Analog Scale (VAS), where 0 represents no pain and 10 represents the worst possible pain. The change in VAS score from baseline to each follow-up time point (6 weeks, 6 months, and 3 years) will be used as the primary endpoint.

#### 3.1.2 Secondary outcomes

Secondary outcomes include the following: biomechanical parameters, assessed by changes from baseline in patellar tracking using dynamic radiography and three-dimensional gait analysis; pain, measured by the Knee Pain Scale (KPS) (26); joint function, evaluated by the Lysholm Knee Scoring Scale (27, 28); kinetic parameters measured by the IDEEA system during stair climbing, including mean power (W) and pulling acceleration (g); spatiotemporal gait parameters measured by the IDEEA system during stair climbing, including walking speed (m/s), step frequency (steps/min), and double support time (ms); and quality of life, assessed by the 36-Item Short Form Health Survey (SF-36) (29–31).

#### 3.1.3 Control of expectation bias

Because the primary outcome is measured using the Visual Analog Scale (VAS), a patient-reported instrument, patients’ treatment expectations may influence their scores and thus introduce expectation bias. To address this issue, we will record patients' treatment expectation scores (on a 0–10 scale) at baseline and compare them between the two groups. If a significant difference exists between the groups, the expectation score will be included as a covariate in sensitivity analyses to adjust for its potential effect on the primary outcome.

### 3.2 Data collection and management

#### 3.2.1 Data collection

To ensure data accuracy and integrity, a dedicated research coordinator will be responsible for collecting all assessment data. Standardized Case Report Forms (CRFs) will be designed, and a dedicated electronic database will be established to systematically record patient demographics, treatment details, and all outcome measures.

#### 3.2.2 Statistical analysis

Data will be analyzed using SPSS software (version 26.0, IBM Corp., Armonk, NY, USA). Continuous variables will be presented as mean ± SD, and categorical variables as frequency (n) and percentage (%). The primary outcome is the change in VAS score from baseline to 6 weeks, and an independent samples *t*-test will be used to compare the change scores between the two groups. For outcomes measured at multiple time points (baseline, 6 weeks, 6 months, and 3 years), a linear mixed-effects model will be employed to account for within-subject correlations and to handle missing data under the missing at random assumption, including group, time, and group-by-time interaction as fixed effects and subjects as random effects.

As this is a non-randomized study, baseline characteristics (including age, sex, body mass index, and baseline VAS score) will be compared between groups, and if any significant imbalance is detected, these variables will be included as covariates in the primary analysis to adjust for potential confounding. Secondary outcomes will be analyzed using independent samples *t*-tests for continuous variables and chi-square tests for categorical variables. Missing data will be handled using multiple imputation for sensitivity analysis. A two-tailed *P*-value < 0.05 will be considered statistically significant for all analyses.

## Patient and public involvement

Patients and/or the public were involved in the design, or conduct, or reporting, or dissemination plans of this research. Refer to the Methods section for further details.
